# Pasireotide Versus Octreotide in Preventing Complications After Simultaneous Pancreas-Kidney Transplantation

**DOI:** 10.3389/ti.2023.11255

**Published:** 2023-06-14

**Authors:** Kaisa Ahopelto, Akseli Bonsdorff, Juulia Grasberger, Marko Lempinen, Arno Nordin, Ilkka Helanterä, Ville Sallinen

**Affiliations:** ^1^ Department of Transplantation and Liver Surgery, University of Helsinki and Helsinki University Hospital, Helsinki, Finland; ^2^ Department of Nephrology, University of Helsinki and Helsinki University Hospital, Helsinki, Finland

**Keywords:** pancreas, postoperative complications, pancreas transplant, somatostatin, pasireotide

## Abstract

In elective pancreatic surgery, somatostatin-analogues pasireotide and octreotide are variably used to reduce postoperative complications, but knowledge on their role in pancreas transplantation is limited. This study compared pasireotide and octreotide for their association with complications after simultaneous pancreas-kidney transplantation (SPK). This retrospective study included consecutive patients undergoing SPK’s from July 2013 to July 2022. Between July 2013 and April 2020, octreotide was administered 0.1 mg s.c. once daily and between May 2020 and July 2022 pasireotide was administered 0.9 mg twice daily, both until third postoperative day. Complications within 90 days postoperatively were collected, and reoperation rate and Comprehensive Complication index (CCI) ≥ 33.7 (morbidity equal to one reoperation) were used as primary outcomes. Of the 213 patients undergoing SPK, 150 patients received octreotide and 63 pasireotide. Baseline characteristics were comparable. Reoperation rate was 25.3% (*n* = 38) and 17.5% (*n* = 11) (*p* = 0.213) and rate of CCI ≥ 33.7 was 40.7% (*n* = 61) and 30.2% (*n* = 19) (*p* = 0.148) in octreotide and pasireotide groups, respectively. When adjusted with donor BMI, pancreas donor risk index, and donor sex, receiving pasireotide translated into OR 0.49 (95% CI: 0.25–0.96 *p* = 0.037) for CCI ≥ 33.7. Pasireotide was independently associated with lower postoperative morbidity within 90 days of SPK compared to octreotide.

## Graphical Abstract

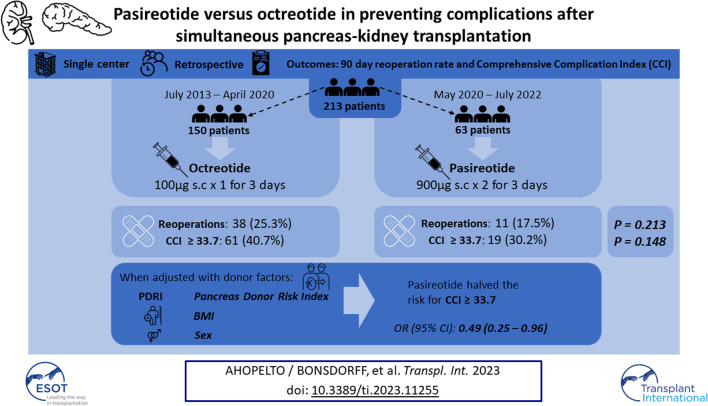

## Introduction

Simultaneous pancreas-kidney transplantation (SPK) offers superior survival over kidney transplantation only (or pancreas after kidney transplantation) to patients with type one diabetes (T1D) and end-stage kidney disease (ESKD) [1, 2]. It improves the quality of life and life-expectancy compared to patients remaining on dialysis [3]. The results of pancreas transplantation have improved over the years [4]. However, the complication burden is still high with as many as 25% of SPK patients undergoing reoperation within 90 days of the transplantation [5]. World consensus guidelines for pancreas transplantation do not discuss the use of somatostatin-analogues [3]. While thrombotic complications are a major cause for graft loss, intra-abdominal infections including pancreatitis and pancreatic fistulas also cause postoperative morbidity.

In elective pancreatic surgery, somatostatin-analogue octreotide has been variably used to prevent pancreatic fistula, but, according to current best evidence, octreotide has no effect on reducing complications, including pancreatic fistula after pancreatic resection [6]. Octreotide has been studied in pancreas transplantation setting, but all the studies are over 15 years old, and its routine use has not gained wide acceptance [7–9].

Pasireotide, another somatostatin-analogue with higher somatostatin receptor affinity and longer half-life, reduced the number of clinically significant pancreatic fistulas compared to placebo after pancreatic resections [10], as well as overall postoperative complications and rate of pancreatic fistulas compared to hydrocortisone in distal pancreatectomies [11] in randomized controlled trials. As noted previously by our group, hyperamylasemia after SPK on postoperative day 1 is a significant risk factor for subsequent morbidity [5]. This finding has been reproduced recently by another study [12], and recent findings have also demonstrated hyperamylasemia and postoperative pancreatitis to have clinical relevance after pancreatic resections [13, 14]. Thus, drugs targeting pancreatic exocrine suppression—such as somatostatin-analogues—may offer a potential mitigation strategy for pancreas graft related complications after pancreas transplantation.

Perioperative octreotide had been routinely used at our center in all SPK since the beginning of our pancreas transplantation program in 2010. Our institutional policy was recently changed to substitute octreotide with pasireotide as of May 2020.

The aim of this study was to compare octreotide and pasireotide for their association with postoperative morbidity after SPK, as well as to assess their association with early postoperative laboratory value trends.

## Materials and Methods

### Patients

This was a retrospective cohort study comparing the association of pasireotide and octreotide with postoperative complications after SPK. Consecutive patients suffering from T1D and ESKD undergoing SPK’s at Helsinki University Hospital, Helsinki, Finland, between 8th July 2013 and 12th July 2022 were included in the study cohort. On 1st of May 2020, our institutional policy was changed from routine perioperative administration of octreotide to pasireotide. Patients undergoing SPK during 8th July 2013 and 30th April 2020 received octreotide 100 ug once daily starting at induction and up to at least 3rd postoperative day (POD). After 1st of May 2020, patients received pasireotide 900 ug twice daily starting at induction and up to the 3rd POD. All the grafts were from donors after brain death (DBD). Immunosuppression, surgical technique, and postoperative care remained similar throughout the study period, and have been described in detail elsewhere [5]. Institutional review board of Helsinki University Hospital approved the study (HUS/155/2021). No ethical board approval was required due to the observational nature of this study.

### Variables Collected

Basic patient and donor demographics were collected. Donor age, donor sex, donor BMI, donor height, donor reason of death, pancreas cold-ischeamia time (CIT), and donor ethnicity were used to calculate pancreas donor risk index (PDRI) for every patient [15]. The PDRI is a continuous risk index where value 1.0 represents an average donor, and higher values represent a higher risk donor.

All postoperative complications occurring before the 90th POD were collected retrospectively from electronic patient records and graded according to the Clavien-Dindo classification [16]. The Comprehensive Complication Index (CCI) was used as an outcome to assess and compare the total cumulative morbidity of the patients [17]. In CCI, raw points are allocated according to the grade of the complication, summed together, and then scaled from 0 to 100. It allows for a much more sensitive comparison of patient outcomes since the cumulative effect of all postoperative complications are captured in the final score. In addition to using CCI as a continuous outcome, a cutoff of 33.7 points, which represents the burden of one reoperation, was used as an outcome in multivariable logistic regression to identify variables associated with higher postoperative morbidity. Pancreas graft associated complications comprised graft pancreatitis, pancreatic fistula/leakage from enteroanastomosis, and peripancreatic fluid collections. Length of hospital stay (LOS) was defined as time in days from the index operation to discharge.

Values of postoperative laboratory tests reflecting pancreatic secretions and inflammation—plasma amylase, drain fluid amylase, and C-reactive protein (CRP) - on each morning up to 7th POD, and laboratory test values reflecting graft function—fasting c-peptide levels, estimated glomerular filtration ratio (eGFR) and HbA1c—up to 180th POD were collected. Plasma amylase values are reported as a multiplication of our institutional upper limit of normal (ULN) to allow for better comparability between centers using different assays. Trends of laboratory values stratified by the type of somatostatin were analyzed to assess for possible differences in exocrine/endocrine suppression. Some cases had missing laboratory test values and multiple imputation (with 10 iterations) was performed with basic patient demographics as dummy variables to account for these missing values. Multiply imputing missing values is associated with smaller bias than excluding cases with missing values [18]. 52/2,130 (2.4%) of c-peptide, 99/1,141 (8.7%) of plasma amylase, 32/852 (3.8%) of HbA1c, 25/852 (2.9%) of eGFR, and 12/213 (5.6%) of donor creatinine values were missing and thus imputed.

### Statistics

Continuous variables are reported as median and interquartile range (IQR) due to nonparametric distribution. Categorical variables are reported as frequencies and percentages. Differences in the distribution of continuous variables between the groups were assessed with Mann-Whitney-U -test and for categorical variables with Chi-squared test. Pre- and intraoperative risk factors for CCI ≥ 33.7 were assessed with logistic regression and a multivariable analysis was performed by including variables with strong univariable association (*p* < 0.15) to a multivariable model constructed with backwards stepwise logistic regression. Somatostatin-analogue variable was forced in to the multivariable model regardless of its univariable association as the aim was to control for the case mix between the cohorts. Variance inflation factors (VIF) were used to assess possible multicollinearity between variables in multivariable analyses. VIF -values under 2.5 are generally interpreted as insignificant correlation between the variables. Odds ratios (OR) with 95% confidence intervals are reported for the uni- and multivariable analyses. In general, a two-sided p-value of < 0.05 was considered statistically significant. All analyses were performed with IBM SPSS v28.

## Results

**FIGURE 1:**
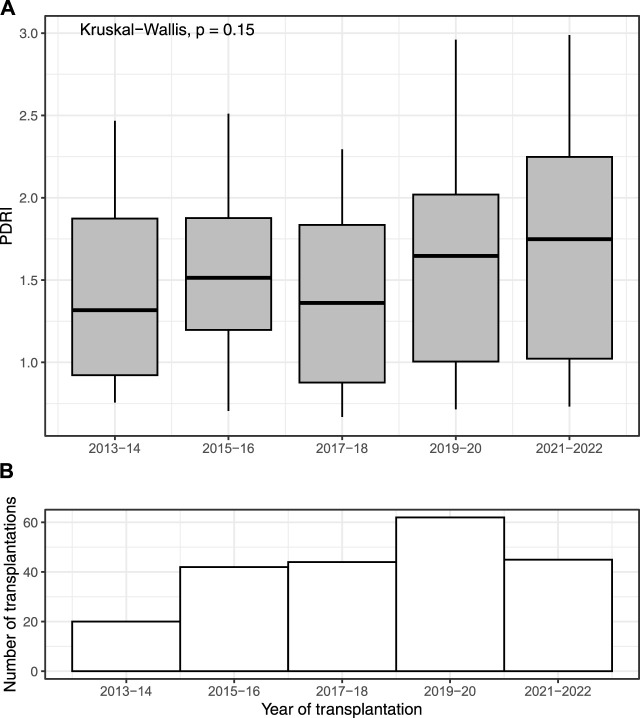
Median (IQR) pancreas donor risk index (PDRI) of 213 patients undergoing simultaneous pancreas-kidney transplantation, reported per year during the study period from 2013 to 7/2022 **(A)**. Number of transplantations per year is reported below **(B)**.

During the study period, 214 patients underwent SPK, of which one from the pasireotide group was excluded due to not receiving the correct drug. The final cohort included 150 patients receiving octreotide and 63 patients receiving pasireotide. The pasireotide and octreotide groups were comparable regarding recipient and donor baseline characteristics, excluding pancreas and kidney cold-ischeamia times (CIT), which were on average 1 h shorter in the pasireotide group, and duration of diabetes, which was on average 8 years longer in the octreotide group (Table 1). PDRI was comparable between the groups (Table 1). Median PDRI levels per 2-year intervals during the study period are illustrated in Figure 1.

**TABLE 1:** Basic demographics of 213 patients undergoing simultaneous pancreas-kidney transplantation, stratified by the type of somatostatin-analogue received perioperatively.

Variable	Octreotide (*n* = 150)	Pasireotide (*n* = 63)	*P* (Chi-squared or Mann-Whitney U)
Age, years, median (IQR)	43 (37–49)	40 (33–48)	0.063
Male sex, n (%)	101 (67.3)	37 (58.7)	0.363
BMI, median (IQR)	24.1 (21.5–27.1)	23.9 (21.7–26.9)	0.769
Donor age, years, median (IQR)	41 (29–50)	45 (28–54)	0.179
Donor, male sex, n (%)	75 (50.0)	31 (49.2)	0.916
Donor BMI, median (IQR)	23.7 (21.9–25.7)	24.5 (21.6–27.3)	0.089
Donor, reason of death, n (%)			0.811
CVA	98 (65.3)	45 (71.4)	
Anoxia	17 (11.3)	7 (11.1)	
Trauma	32 (21.3)	10 (15.9)	
Other	3 (2.0)	1 (1.6)	
> 2 HLA-AB mismatch (*n* = 138)	93 (62.0%)	45 (71.4%)	0.217
> 1 HLA-DR mismatch (*n* = 120)	84 (56.0%)	36 (57.1%)	0.754
Cold ischeamia time, pancreas (hours)	7.78 (6.27–8.77)	6.75 (5.42–7.92)	0.001
Cold ischeamia time, kidney (hours)	9.74 (7.74–10.77)	8.27 (5.70–9.75)	<0.001
Duration of diabetes in years, median (IQR)	34 (28–40)	26 (23–36)	<0.001
Duration of dialysis before transplantation, months, median (IQR)	13 (8–19)	13 (8–21)	0.604
PDRI, median (IQR)	1.48 (1.00–1.89)	1.74 (0.96–2.26)	0.097

Abbreviations: IQR, inter-quartile-range; BMI, body-mass-index; CVA, cerebrovascular attack; HLA, human leukocyte antigen; PDRI, pancreas donor risk index.

### Postoperative Complications and Outcomes

The frequency of individual postoperative complications is presented in Table 2. Hemorrhagic complications (Clavien-Dindo grade IIIa or worse) were the most common, occurring in 38 (17.8%) individual patients, followed by intra-abdominal fluid collections, which occurred in 30 (14.1%) individual patients. Only two pancreas graft thromboses were observed, both of them partial and successfully treated with anticoagulants.

**TABLE 2:** Frequency of postoperative complications in the whole cohort and the somatostatin -groups of 213 patients undergoing simultaneous pancreas-kidney transplantation.

Complication	All (*n* = 213)	Octreotide (*n* = 150)	Pasireotide (*n* = 63)	*p*-value
Hemorrhage, region of graft pancreas, CD IIIa or worse	29 (13.6%)	20 (13.3%)	9 (14.3%)	0.853
Hemorrhage, region of graft kidney, CD IIIa or worse	10 (4.7%)	6 (4.0%)	4 (6.3%)	0.459
Pancreas graft thrombosis, any grade	2 (0.9%)	1 (0.7%)	1 (1.6%)	0.525
Graft pancreatitis, any grade	19 (8.9%)	17 (11.3%)	2 (3.2%)	0.057
Pancreatic fistula, CD IIIa or worse	3 (1.4%)	3 (2.0%)	0	0.258
Ileus, any grade	15 (7.0%)	11 (7.3%)	4 (6.3%)	0.798
Bowel perforation, CD IIIb or worse	2 (0.9%)	1 (0.7%)	1 (1.6%)	0.525
Wound dehiscence	13 (6.1%)	10 (6.7%)	3 (4.8%)	0.596
Peripancreatic fluid collection, CD IIIa or worse	9 (4.2%)	7 (4.7%)	2 (3.2%)	0.621
Perirenal fluid collection, CD IIIa or worse	21 (9.9%)	20 (13.3%)	1 (1.6%)	0.009
Hydronephrosis, CD IIIa or worse	16 (7.5%)	15 (10.0%)	1 (1.6%)	0.034
Unexplained fever, CD II or worse	47 (22.1%)	30 (20.0%)	17 (27.0%)	0.262

*p*-values calculated for octreotide vs. pasireotide with chi-squared test. CD, Clavien-Dindo (grade).

The reoperation rate up to 90th POD was 25.3% (*n* = 38) in the octreotide group, and 17.5% (*n* = 11) in the pasireotide group, but this difference was not statistically significant (*p* = 0.213). These results would translate into absolute risk reduction (ARR) of 7.8% and a number needed to treat (NNT) of 13 to avoid one reoperation. The most prevalent reason for reoperation in the whole cohort was hemorrhage [25/49 (51.0%)], followed by pancreas graft associated complications [13/49 (26.5%)], and postoperative ileus [5/49 (10.2%)]. No significant differences were observed for these reasons of reoperation between the groups (Table 3). Four (1.9%) pancreas grafts were lost during the 90-day postoperative period due to persistent intra-abdominal infections, and all occurred in the octreotide group.

**TABLE 3:** Postoperative outcomes up to 90th postoperative day of 213 patients undergoing simultaneous pancreas-kidney transplantation, stratified by the type of somatostatin-analogue received perioperatively.

Variable	Octreotide (*n* = 150)	Pasireotide (*n* = 63)	*P* (Chi-squared or Mann-Whitney U)
Length of hospital stay (d), median (IQR)	16 (13–24)	14 (10–19)	0.009
Reoperation, n (%)	38 (25.3)	11 (17.5)	0.213
Pancreas graft loss, 90 days, n (%)	4 (2.7)	0	0.191
Reoperation due to hemorrhage, n (%)	18 (12.0)	7 (11.1)	0.854
Reoperation due to pancreas graft associated complication, n (%)	10 (6.7)	3 (4.8)	0.759
Reoperation due to postoperative ileus, n (%)	3 (2.0)	2 (3.2)	0.605
Comprehensive Complication Index, median (IQR), 30 days	29.6 (20.9–42.6)	29.6 (20.9–33.7)	0.833
Comprehensive Complication Index, median (IQR), 90 days	29.6 (20.9–43.4)	29.6 (20.9–34.8)	0.434
CCI ≥ 33.7, n (%)^a^	61 (40.7)	19 (30.2)	0.148
CCI ≥ 47.7, n (%)^a^	21 (14.0)	6 (9.5)	0.370
Drainage of intra-abdominal fluid collection, n (%)	26 (17.3)	3 (4.8)	0.015
Organ space or deep SSI, n (%)	17 (11.3)	3 (4.8)	0.133
Pancreas associated complication, Clavien-Dindo II or worse, n (%)	40 (26.7)	12 (19.0)	0.259
PONV, DGE, ileus	24 (16.0)	7 (11.1)	0.265

CCI, comprehensive complication index; SSI, surgical site infection; PONV, postoperative nausea and vomiting; DGE, delayed gastric emptying.

^a^ CCI ≥ 33.7 equals cumulative morbidity of one reoperation, ≥47.7 of two reoperations.

**FIGURE 2:**
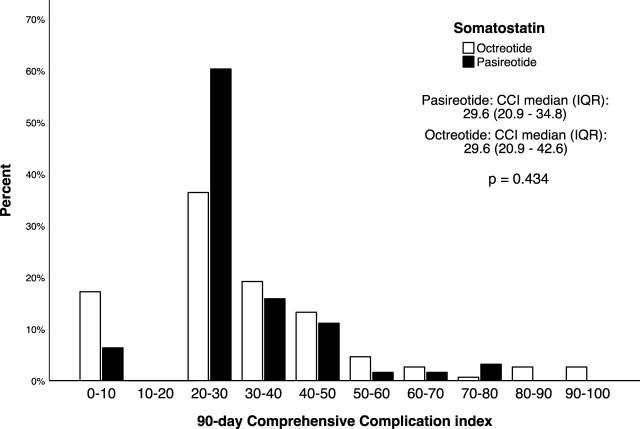
The distribution of Comprehensive Complication index in 213 patients undergoing simultaneous pancreas-kidney transplantation, stratified by the type of perioperative somatostatin-analogue received.

The median (IQR) CCI was similar between the groups at both 30th and 90th POD timepoint with little difference between the timepoints demonstrating that most of the severe complications occurred during the first 30 postoperative days (Table 3). The 90-day CCI distribution stratified by somatostatin-analogue received is presented in Figure 2. The length of initial hospital stay was statistically significantly longer in the octreotide group, median 16 (IQR: 13–24) vs. median 14 (IQR: 10–19), *p* = 0.009 (Table 3). In addition, the incidence of intra-abdominal fluid collections requiring radiological intervention was significantly higher in the octreotide group, 26 (17.3%) vs. 3 (4.8%), *p* = 0.015. Other studied outcomes were similar between the groups (Table 3).

Pre- and intraoperative risk factors for the morbidity of one reoperation (CCI ≥ 33.7, *n* = 80, 37.6%) are presented in Table 4. Recipient age, BMI, estimated blood loss, duration of diabetes, and pancreas CIT had *p*-values over 0.150 in univariable analyses with morbidity, and were omitted from the backwards stepwise logistic regression. According to the final multivariable analysis, the probability for CCI ≥ 33.7 was lower for patients receiving pasireotide when adjusted with the identified risk factors PDRI, donor BMI, and donor sex, 0.49 (95% CI: 0.25–0.96), *p* = 0.037. VIF -values for somatostatin, PDRI, donor BMI and donor sex in this multivariable model were 1.08, 1.15, 1.11, and 1.03, respectively, showing neglibile multicollinearity between the variables.

**TABLE 4:** Univariable and multivariable analysis of pre- and intraoperative risk factors for one reoperation’s morbidity (CCI ≥ 33.7) (*n* = 80, 37.6%).

Variable	Univariable		Multivariable	
	OR (95%CI)	*p*	OR (95%CI)	*p*
Age (y)	1.02 (0.98–1.05) per increase of 1 year	0.367		
BMI (kg/m^2^)	0.97 (0.88–1.06) per increase of 1 kg/m^2^	0.469		
Duration of diabetes, years	1.00 (0.97–1.03) per increase of 1 year	0.954		
Male sex	1.02 (0.57–1.81)	0.960		
PDRI	1.92 (1.17–3.15) per increase of 1	0.010	1.92 (1.11–3.33) per increase of 1	0.020
Donor age (y)	1.03 (1.01–1.05) per increase of 1 year	0.017		
Donor BMI (kg/m^2^)	1.13 (1.03–1.24) per increase of 1 kg/m^2^	0.009	1.11 (1.00–1.23) per increase of 1 kg/m^2^	0.049
Donor male sex	1.65 (0.94–2.88)	0.081	1.80 (0.99–3.24)	0.051
Estimated blood loss (mL)	1.03 (0.94–1.14) per increase of 100 mL	0.499		
Nontraumatic donor death	1.32 (0.65–2.67)	0.449		
Cold ischeamia time, pancreas	1.09 (0.92–1.29) per increase of 1 h	0.310		
Cold ischeamia time, kidney	1.10 (0.97–1.24) per increase of 1 h	0.140		
Pasireotide (compared to octreotide)	0.63 (0.34–1.18)	0.150	0.49 (0.25–0.96)	0.037

Variable was entered into multivariable analysis if univariable association *p* < 0.15, backwards stepwise method was used.

Abbreviations: BMI, body-mass-index; CCI, comprehensive complication index; PDRI, pancreas donor risk index.

### Laboratory Test Trends

**FIGURE 3:**
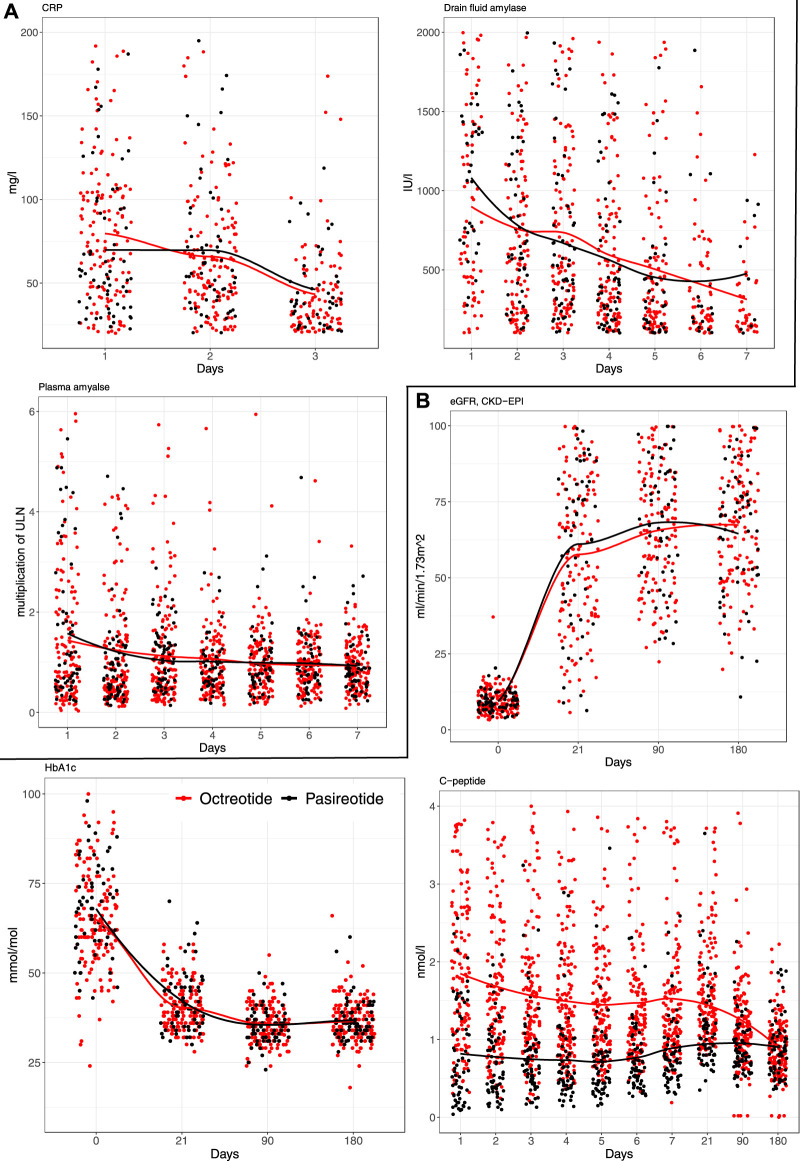
Trend curves for **(A)** early and **(B)** late postoperative laboratory values after 213 simultaneous pancreas-kidney transplants, stratified by the type of somatostatin-analogue received.

To assess the association of pasireotide and octreotide on pancreatic secretions, trend lines stratified by the type of somatostatin received were drawn for plasma amylase, drain fluid amylase, and CRP (Figure 3A). In general, the trend curves were declining in nature with the highest values occurring on the earliest POD’s. No statistically significant differences were observed for amylases or CRP. Other laboratory variable trends reflecting graft function (eGFR, HbA1c, and c-peptide) are presented in Figure 3B. Interestingly, while significant difference in c-peptide levels during the first postoperative week was observed, this difference leveled off at the 180-day timepoint.

## Discussion

SPK predisposes patients to high risk for postoperative morbidity with a reoperation rate close to 25% [5, 19]. While graft thrombosis is commonly reported to account for the majority of pancreas graft loss, other complications more related to the exocrine pancreas function—like graft pancreatitis, anastomotic leaks, pancreatic fistulas and intra-abdominal infections—seem to contribute significantly to the overall morbidity [20, 21]. In this present study, overall reoperation rate was 23%, with postoperative hemorrhagic complications accounting for roughly half of the reoperations, followed by graft pancreatitis/infection in one-fourth of the cases. Postoperative graft loss rate was 1.9%, and all were due to persistent intra-abdominal infections. Additionally, in this retrospective study, pasireotide was independently associated with lower postoperative morbidity after SPK compared to octreotide.

While great efforts have been made to reduce complications in elective pancreas surgery, few trials include patients undergoing pancreas transplantation. Somatostatin-analogues have been used in pancreatic surgery to reduce the risk of complications, especially pancreatic fistulas [10, 11, 22]. While several RCTs assessing the efficacy of octreotide exists, and meta-analysis of these RCTs show no effect [6], only two RCTs assessing pasireotide exists, both of which show benefit.

There are three randomized controlled trials comparing octreotide to no treatment in pancreas transplantation setting [8, 9, 23], but all of them are over 15 years old and significantly underpowered due to small sample sizes.

The first randomized study of a somatostatin-analogue in pancreas transplant setting, by Stratta et al. in 1993, compared 13 patients that received octreotide 100 µg twice daily to 12 patients that received no somatostatin treatment [7]. Octreotide was initiated after transplantation and continued for 8 (±4) days. Octreotide reduced drain fluid amylase output, but there were no significant differences between the groups in patient or graft survival, infection, or surgical complications. In 1998, a study with 10 patients receiving perioperative octreotide 100 µg three times daily and seven patients receiving no treatment was conducted [8]. The patients in the octreotide group had no complications compared to the group receiving no somatostatin-analogue where one patient had a bladder leak and two developed intra-abdominal infections. Patient and graft survival were similar in both groups. In 2005, Hesse et al. reported no difference between 20 patients receiving perioperative octreotide 100 µg three times dailys compared to 20 patients receiving no treatment in terms of formation of pancreatic fistula (2 vs. 0). As octreotide interferes cyclosporine metabolism and possibly other immunosuppressive therapy as well and is costly, the study concluded that prophylactic treatment with octreotide cannot be recommended.

An obvious limitation of these existing studies is small sample size, the largest study recruiting 20 patients per arm, leading to underpowered results and difficulties in drawing conclusions. Of note, the first two studies from the last century used bladder drainage technique instead of enteric drainage, and as such the results might not be generalizable to the contemporary era.

The levels of serum and intra-abdominal amylase was shown to be lower in the group receiving octreotide in these previous studies [8]. In our study, serum and drain fluid amylase levels were comparable between the groups, and receiving pasireotide did not seem to translate into stronger exocrine suppression. As no control group was available, it is difficult to assess the exocrine suppressive effect of these somatostatins. As noted in previous studies [5, 12], early hyperamylasemia is a significant risk factor for subsequent morbidity after SPK, and interventions mitigating it—such as somatostatin-analogues—could be of interest [5]. Interestingly, c-peptide levels were significantly lower throughout the first 7 POD’s in the pasireotide group but leveled off during the 180-day follow up, and did not seem to associate with adverse events.

To the best of our knowledge, there are no other studies assessing pasireotide in pancreas transplantation setting. Pasireotide seems to be a safe alternative for octreotide and was independenctly associated with reduction of severe postoperative complications when compared to octreotide. Reoperation rate was 17.5% in the pasireotide group compared to the 25.3% in the octreotide group. Patients in the pasireotide group had a significantly lower incidence of intra-abdominal collections requiring radiological intervention (17.3% vs. 4.8%) and spent on average 2 days less in the hospital. The shorter hospital stay could be confounded by an overall trend to shorter hospital stays over the years, as the patients in the pasireotide group were operated later during the study period. In addition, pancreas CIT was statistically significantly shorter in the pasireotide-group, but this finding did not translate into an association with morbidity. This may be explained by the fact that median CIT was relatively short in both cohorts (7.8 h in the octreotide-group, 6.8 h in the pasireotide-group), and previous studies have identified CIT exceeding 12 h to associate with heightened morbidity [24]. When adjusted with PDRI, donor BMI, and donor sex to control for case-mix, receiving pasireotide translated into OR 0.49 for high postoperative morbidity compared to octreotide. On another note, no clinically meaningful outcomes favored octreotide in the comparisons. No significant difference in early amylase and CRP, or post-transplant eGFR or HbA1c levels was observed between the groups.

### Limitations

There are several limitations to our study. Use of octreotide was introduced at the beginning of our SPK-program in 2010, and it was adapted and modified from other existing protocols. Partly due to the lack of evidence supporting octreotide use and the promising results from the pancreatic surgery RCTs by Allen et al. in 2013 and Tarvainen et al. in 2020 our protocol was changed [10, 11]. This is a retrospective analysis of the short-term results of this change. This was not planned as a study and thus lacks a control group. All our patients received a somatostatin-analogue and based on these results, we do not know the incidence of pancreas graft related complications if a somatostatin-analogue had not been used. The patients in the octreotide treatment group received a significantly smaller dose than in all other studies and one might argue that octreotide 100 µg daily is not comparable to pasireotide 900 µg twice a day, rather closer to no treatment. While generally unadvisable, CCI was dichotomized due to a relatively small sample size and its discrete distribution, and this might introduce optimism to the multivariable estimate of pasireotide effect size. Due to the retrospective setting, controlling for confounders is subpar and no causality can be concluded. Finally, while the study cohort is relatively large for a cohort of pancreas transplantations, the statistical analyses suffer from lack of power and most likely type 2 error is present. In order to have a 80% chance of detecting the reduction in reoperation rate reported in this study (from 25.3% to 17.5%), as significant at the 5% level, 862 patients would have been required.

## Conclusion

Pasireotide is safe to use for patients receiving SPK transplant and its use was independently associated with reduced severe complications up to 90 days post-transplantation. Further prospective randomized study in larger cohorts is warranted but may be difficult to carry out due to relatively large number of patients required for statistical power.

## Data Availability

The datasets presented in this article are not readily available because data is not available due to the regional legislation. Requests to access the datasets should be directed to ville.sallinen@helsinki.fi.

## Abbreviations

ARR, absolute risk reduction; BMI, body mass index; CCI, comprehensive complication index; CRP, C-reactive protein; DBD, donor after brain death; ESKD, end-stage kidney disease; IQR, interquartile range; NNT, number needed to treat; POD, postoperative day; RCT, randomized controlled trial; SPK, simultaneous pancreas-kidney transplantation; TID, Type 1 diabetes mellitus; ULN, upper limit of normal.
